# A Novel IoT Based Positioning and Shadowing System for Dementia Training

**DOI:** 10.3390/ijerph18041610

**Published:** 2021-02-08

**Authors:** Lun-Ping Hung, Weidong Huang, Jhih-Yu Shih, Chien-Liang Liu

**Affiliations:** 1 Department of Information Management, National Taipei University of Nursing and Health Sciences, Taipei 111219, Taiwan; kiel.hung@gmail.com (L.-P.H.); shih10036781@gmail.com (J.-Y.S.); 2 TD School, University of Technology Sydney, 2007 Ultimo, Australia; weidong.huang@uts.edu.au; 3 Dementia Center, Taipei City Hospital, Taipei 10065, Taiwan

**Keywords:** short-term memory, cognitive training, compensatory memory aids, speech shadowing, indoor localization

## Abstract

A rapid increase in the number of patients with dementia, particularly memory decline or impairment, has led to the loss of self-care ability in more individuals and increases in medical and social costs. Numerous studies, and clinical service experience, have revealed that the intervention of nonpharmacological management for people with dementia is effective in delaying the degeneration caused by dementia. Due to recent rapid developments in information and communications technology, many innovative research and development and cross-domain applications have been effectively used in the dementia care environment. This study proposed a new short-term memory support and cognitive training application technology, a “positioning and shadowing system,” to delay short-term memory degeneration in dementia. Training courses that integrate physical and digital technologies for the indoor location of patients with dementia were constructed using technologies such as Bluetooth Low Energy, fingerprint location algorithm, and short-range wireless communication. The Internet of Things was effectively applied to a clinical training environment for short-term memory. A pilot test verified that the results demonstrated learning effects in cognitive training and that the system can assist medical personnel in training and nursing work. Participants responded with favorable feedback regarding course satisfaction and system usability. This study can be used as a reference for future digital smart cognitive training that allows observation of the performance of patients with dementia in activities of daily living.

## 1. Introduction

Memory loss is a natural process of aging, but it greatly affects daily life and work. Dementia is regarded as a symptom of memory loss. It is an irreversible disease, and its prevalence increases with age. According to the Global Action Plan on the Public Health Response to Dementia initiated by the World Health Organization, a total of 47 million people had dementia in 2015, accounting for approximately 5% of the global aging population. The plan estimated that the number of people with dementia will increase to 75 million in 2030 and 132 million in 2050 [1]. Cognitive decline in dementia largely begins in the hippocampus and may causes disability and dependence among older adults. Common dementias include dementia with Lewy bodies, frontotemporal lobe degeneration, and Alzheimer’s disease (AD). Different types of dementia have different initial symptoms. Among these dementias, AD is the most common and usually begins with a degeneration in short-term memory. Since the cognitive functions of people with AD have been impaired, they encounter difficulty in remembering things contacted in the short term and thus cannot form recent memories. In addition, the short-term memory loss caused by dementia may lead to a sudden loss of memory or concentration as well as personality changes. The entire course of dementia can be divided into mild cognitive impairment (MCI), mild, moderate, and severe dementia. Moreover, the loss of memory is continuous. Impairment of short-term memory affects a patient’s work ability and family relationships, with serious effects on patients, family members, caregivers, communities, and society.

Relevant studies have confirmed that cognitive training can effectively improve the cognitive functions of patients with dementia and have an indirect positive effect on functional deficits in activities of daily living [2,3]. As an example of short-term memory training methods, speech shadowing exercise asks patients to imitate and repeat a spoken stimulus as accurately as possible [4]. Further, Kang et al. developed a series of paper-and-pencil-based cognitive training programs with different levels of difficulties in order to improve subskills of memory, language, attention, visuospatial function, and calculation [5]. The authors conducted a user study and found that participants who received regular training of the programs demonstrated significant improvements in those subskills.

Furthermore, a digital age has emerged, with rapid development of digital technologies and the introduction of new digital applications. To escape conventional thinking and care models and to solve the problem of declining birth-rates, technology must be integrated into the lives of older adults. In recent years, information and communications technology (ICT) has been continually upgraded. In particular, the use of the Internet of Things (IoT) in mobile learning has achieved favorable results. The integration of IoT and mobile learning with related technologies is expected to achieve excellent results. On the basis of the IoT in the health care industry, this study proposed a short-term memory multi-sensory learning cognitive training model including Bluetooth low energy (BLE), a fingerprint location algorithm, and received signal strength indication (RSSI) integrated with digital, multimedia rehearsal materials and blended into context, with the aim of developing a set of short-term memory cognitive training courses suitable for patients with early dementia experiencing short-term memory loss. We constructed digital short-term memory training classes and a mobile positioning algorithmic mechanism to integrate the real-time virtual and real cognitive training technology, so the patients of short-term memory loss will be able to achieve basic self-life governing ability with the help of IoT technology and compensatory memory assistance. Furthermore, with the recording and storage functions of the information system, therapists, and practitioners are able to grasp key factors, such as cognitive changes and the learning willingness of older adults with dementia, which provides clinical decision-making support to medical-related units for improved disease control and treatment effects.

This paper is the extended version of the conference paper [6]. This extended version included a literature review of more related works, more detailed information about our methodology and system and a complete system testing report. The remainder of the paper is organized as follows: In Section 2, we describe the concept of the process of memory, application of memory training and indoor positioning methods. Section 3 presents our methodology. A practical example is used to illustrate the application of the new short-term memory support and cognitive training application technology, including training process, calculation mechanism, course module, and management platform. Section 4 introduces the experimental simulation results for positioning. It also summarizes the system testing results with end users (using interviews and questionnaires). Finally, Section 5 presents our conclusions.

## 2. Literature Review

Drugs for the treatment of dementia have not been able to prevent damage or to restore brain cells, but medical and nonpharmacological treatments can delay disease development. Nonpharmacological treatment includes cognitive training, reality orientation therapy, validation therapy, reminiscence therapy, and horticultural therapy. Their purpose is to ameliorate a patient’s symptoms or delay the progression of the disease, enhance the patient’s quality of life, and reduce the burden on caregivers. Cognitive training is considered a means to improving memory. Since the brain controls cognitive functions, repetitive training can strengthen brain cell connections and thus improve attention, memory, language, and executive functions, and it can have an indirect positive effect on functional defects in activities of daily living. Using the aforementioned therapies in appropriate treatment can improve the social and professional integration of patients [2]. Cognitive training combined with E-learning has yielded favorable results. Its application in short-term memory training must primarily consider memory process, strategy application, the ICT capability, and the combined application of the aforementioned technology.

### 2.1. Human Memory Process and Memory Strategy

**Figure 1:**
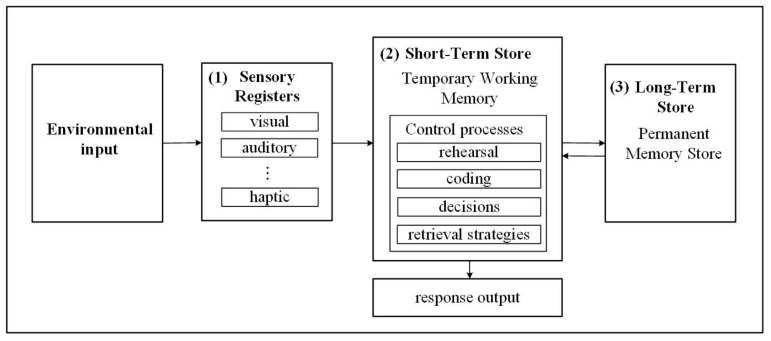
Information processing flow.

Memory is a mental function used to retain and retrieve past experience for daily use. The process of memory consists of encoding, storage, and retrieval [7]. Atkinson and Shiffrin (1968) divided memory into three storage stages [8], namely, sensory store, short-term store, and long-term store. The information processing flow is shown in Figure 1. (1) Sensory store refers to the memory type in which information is unprocessed and remains for less than 1 s after the information is received by the senses; it is also known as immediate memory. (2) The short-term store is the stage of processing most of the information. Through the hippocampus, crucial parts of the short-term memory can be preserved to form long-term memory, but capacity is relatively limited to approximately 15–30 s. (3) Long-term memory refers to storage that lasts not a few seconds but a lifetime. The current research asserts that memory capacity at this stage is unlimited. Short-term memory is a mental workspace with limited capacity, the function of which is short-term storage and execution of information. In addition, it substantially affects human cognitive activities such as mathematical calculation, reading comprehension, problem solving, and inference, all of which are vital indicators for evaluating differences in cognitive functions.

In addition to daily diet and daily routine adjustments, strengthening the brain is essential in preventing hippocampus degeneration and improving short-term memory. In clinical trials, numerous memory training methods have proved to be effective. Klingberg confirmed that the cognitive functions of the brain, such as short-term memory, information processing speed, and abstract reasoning ability, can be improved through training, particularly through an online digital training design that can effectively enhance cognitive abilities [9]. In 1965, LaBerge and Winokur noted that shadowing exercise can enhance short-term memory [10]. Later, Sternberg and Sternberg proposed the strategy of using rote learning to strengthen short-term memory [11], as follows: (1) starting with attention, and focusing on the information that needs to be memorized; (2) using multiple codes simultaneously to convert the image code produced by the visual organ into the sound code produced by the auditory organ; (3) chunking, which involves gathering multiple small chunks in the information into a big chunk, which is then used as the memory unit; (4) paying attention to the characteristics of the information and thus deepening the impression of the information; and (5) rehearsal. In 2014, experts from the American Translators Association proposed some segmented interpretation training methods [12], such as shadowing and shadowing with a twist to improve short-term memory. Among them, the methods of shadowing and shadowing with a twist have proved their training effects in various studies [9,10]. Many subsequent studies were expected to develop new or mixed training models based on these methods to effectively improve memory training and learning effects.

### 2.2. Information and Communications Technology That Supports the Application and Development of Memory Training

Memory training is a commonly used method in cognitive training. The goal is to adapt to, understand, or reduce the effect of memory impairment on daily life through various strategies and techniques, such as designing memory aids or teaching memory-assisting skills. The focus of memory training is to prevent or slow further degeneration, not to restore memory [13,14].

Galante et al. conducted a single-blind randomized controlled trial of computer-assisted cognitive rehabilitation of 11 patients with AD and MCI [15]. The computer training lasted for four weeks, and each training session was 60 min in duration. The results indicated that computer-assisted cognitive rehabilitation can delay the cognitive decline of patients with AD and MCI. Chang et al. used an application to assist the memory retrieval of patients with traumatic brain injury, and used memory training flashcards to prompt the recall of memories [16]. The training was performed using a question and answer method, and the questions were linked with the daily life events of the user and were automatically generated from the memory storage of the application. Each question included four key factors identified by the authors, namely people, location, time, and activity. Patients answered the questions on smartphones. The purpose of the study was to use flashcards for active memory training [16]. Munoz-Montoya et al. proposed an augmented reality (AR) technology based on simultaneous localization and mapping to evaluate short-term spatial memory [17]. The study invited 55 participants, and they were divided into the ARGroup (using AR to learn the location of virtual objects in the real environment) and the NoARGroup (viewing photos to learn the location of objects). The participants in the ARGroup outperformed those in the NoARGroup in memorizing objects and their positions [17]. Rohrbach et al. used AR to provide activities of daily living support for patients with AD [18]. In their crossover study, a head-mounted Microsoft HoloLens was used to verify the feasibility and usability of AR that supported patients with AD in tea brewing activities. However, the application had no apparent positive effect on patient performance. The surmised reason was that the AR application only provided seven-step prompts and lacked more detail, which confused the patients. Therefore, the provision of more detailed content is crucial to improving the completion rate of patients [18].

In summary, relevant studies have confirmed that cognitive training can effectively improve the cognitive functions of patients with dementia. However, no virtual interactive memory training model using IoT has been developed for MCI in Taiwan or abroad. Therefore, a set of short-term memory cognitive training courses suitable for older adults was designed, and a multi-sensory learning environment was constructed through the IoT to increase motivation and willingness for continuous engagement with the courses to achieve cognitive training effects.

### 2.3. Indoor Positioning System

**Figure 2:**
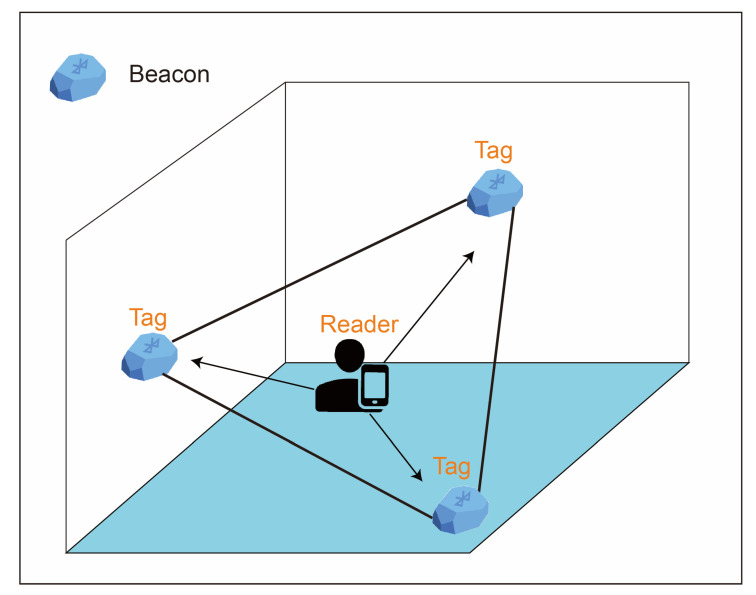
Triangulation.

**Figure 3:**
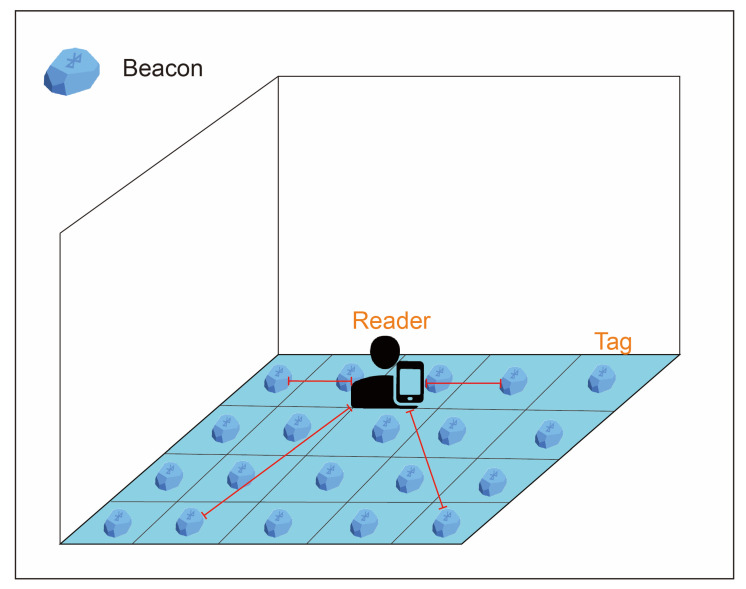
Proximity.

A real-time location system based on wireless sensor networks is used to locate and track targets in real-time through received information. The modes of the real-time location system can be divided into time of arrival, time difference of arrival, angle of arrival, and received signal strength index (RSSI). RSSI positioning does not require special equipment or strict network synchronization, which renders it particularly suitable for large-scale wireless sensor networks. Li et al. classified three positioning modes or methods of RSSI for indoor use [19]. The first was the triangulation method, shown in Figure 2, and is the most basic positioning method of the three. RSSI values differ according to the distance between the tag and the reader. With three readers placed in the environment, the RSSI values of the tags are the signals received by the readers. Users can use the signals to calculate the location according to the distance decay mode. However, various objects often exist in a general environment. These objects, of different materials, cause refraction and scattering of the signal, resulting in multiple paths during signal transmission, thus causing the triangulation method to fail and the error value to increase. Therefore, this positioning method has gradually been abandoned, and most researchers have begun to use other positioning methods or to integrate other sensing elements to increase positioning accuracy. The second method is the proximity deployment method, depicted in Figure 3. The environment is divided into multiple blocks, and in each block is placed an active tag as a reference tag. The reference tag continues to send a signal value for the reader to receive, and the reader matches the received signal value to the signal value of the block. When the target wearing the tag enters the environment, the reader receives both the target and reference tag signals and compares the signals to identify the reference value closest to the target, and then determines the area where the target is located. This positioning method requires a large number of reference tags to be deployed in the environment. With the expansion of the application environment, the number of reference tags deployed in the environment increases, and so does the construction cost. Therefore, the proximity method is more suitable for use in a small environment. In constructing the environment, the positioned location is more accurate if area distinction is more refined.

**Figure 4:**
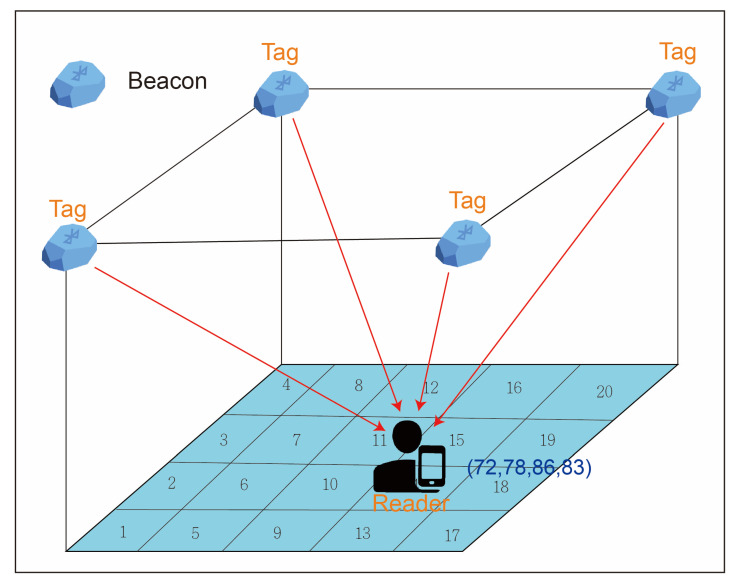
Location fingerprinting.

The third method is the fingerprint location algorithm (see Figure 4). A fingerprint location algorithm requires pre-investigation of each reference tag. The environment layout is similar to that of the proximity method; the key difference is that the fingerprint location algorithm does not require continuous deployment of reference tags. The signal value of the reference tag in the environment is recorded in the database in advance. When the positioning target carrying the active tag moves in the environment, the reader reads the signal value of the tag and compares it with the value of the virtual reference tag in the database to identify the reference tag nearest to the target and determine the target’s location. This method can reduce the installment cost because no tags are needed in the environment. However, the signal value of the reference tag stored in the database is not revised as the environment changes. If the environment changes, the signal value of the reference tag must be rerecorded to avoid positioning errors.

The actual environment designed for this study required participants to move using different walking paths, thus, the linear signal transmission states of the reader and the tag could not be constantly maintained. Therefore, triangulation positioning was unsuitable for use in the environment. Since the proximity method requires that numerous tags be used as reference points (RPs) in the environment, it was infeasible due to the cost of actual construction. In consideration of deployment cost and feasibility, a smart device was used to read the RSSI signal value in the environment to determine the location of the target. Moreover, a smart device can learn the current range of the participants and the location of the training point according to the results of mobile computing, which could assist the memory training of the participants.

### 2.4. Machine Learning Integrated with Positioning Methods

Using only radio frequency for positioning cannot reflect the actual position due to factors such as diffraction and refraction in the environment. Bluetooth devices with a broadcast frequency of 2.400–2.485 GHz can provide an example. When multiple devices of the same frequency band are installed near the receiver, the devices are prone to conflict with each other, and the receiver is unable to receive signals correctly. Therefore, machine learning algorithms have been added in many studies to estimate the final position with improved accuracy. For example, Stavrou et al. [20] used a BLE beacon as an ensemble filter for indoor positioning. In the study, a fingerprint positioning combined with random forest algorithm was used in a retail store positioning application, and the positioning error results were 2 m–2.5 m. The authors noted that interference in the environment should be evaluated when performing indoor positioning, and the interference should be eliminated to achieve more satisfactory results. In the future, WiFi technology can be integrated to explore the effectiveness of positioning [20]. Ho and Chan [21] proposed a decentralized BLE-based positioning protocol. This algorithm training process removed the centralized server and manual signal training process. Its function was based on the beacon’s modification capability, which enabled the beacon to broadcast and scan for signals simultaneously to achieve automatic training, thereby ensuring that the parameters in the model were current. In the experiment, 40 RPs were calculated using K-nearest neighbors (KNN), and the results revealed that the positioning performance was optimal when *k* = 2, with a positioning error of 1.5 m [21]. Subedi and Pyun [22] proposed a method that combined a fingerprinting localization with weighted centroid localization to create a weighted K-nearest neighbors (WKNN) algorithm. Compared with conventional fingerprinting localization approaches, the technology proposed by the study reduced the number of fingerprint RPs required by 49.23%, and positioning error was reduced to approximately 1 m [22].

Cannizzaro et al. integrated triangulation and a fingerprinting localization with machine learning regression methods, including KNN, multilayer perceptron, and support vector machine, and performed analysis and comparisons for an industrial environment [23]. He deployed three to four beacons in two real situations to understand possible positioning changes in different situations. The KNN algorithm was used to test different *k* values to identify the data that produced the lowest median error in all test datasets. Using *k* = 3 had the optimal positioning results. For the multilayer perceptron method, the lowest error was found when the three hidden layers contained 8, 8, and 6 neurons and when the rectified linear unit activation function was used. In the support vector machine method, the lowest median error was based on the radial basis function kernel γ = 0.01, ε = 0.02, and C = 1. The final results suggested that the fingerprinting localization outperformed the triangulation method. In addition, KNN had the simplest deployment method and fewest parameters of the machine learning methods, and it was therefore chosen as the primary algorithm. Ke et al. used a fingerprinting localization algorithm to construct a BLE beacon-based location system for smart home power management [24]. The study integrated the fingerprinting localization algorithm with WKNN for positioning operation. It also used a mean filter to remove noise for improved signal stability. The mean filter was a linear filter that calculated the mean value from the sum of the measured signals, which removed signal values with larger error and had a low-pass effect [24]. In accordance with the literature reviewed, as a tool for the positioning module, the WKNN method with the smallest positioning error was adopted for this study, and a mean filter was added to reduce signal noise (to an extent).

## 3. Method

This study proposed a new short-term memory support and cognitive training application technology named a “positioning and shadowing system.” The system employed a short-term memory training model using an indoor positioning system to integrate physical and digital learning content. Based on perception and dementia compensatory memory aids, the system was combined with digital multimedia short-term memory and rehearsal materials to create a multi-sensory training environment for visual, auditory, and haptic perceptions. In addition, the intervention with the information system enabled the identification of key factors, such as cognitive change in older adults with dementia and their learning willingness, to achieve satisfactory disease control and treatment effects. The positioning method, training process, calculation mechanism, course module, and management platform designed for the study are introduced separately.

### 3.1. Positioning Method

The positioning method included two execution programs. First was a physical and digital learning content training model that integrated with an indoor positioning system. Second was a content-driven process design of a fixed-point interactive course. The digital content in the study was based on the digital content generated by the location point, as detailed later.

**Figure 5:**
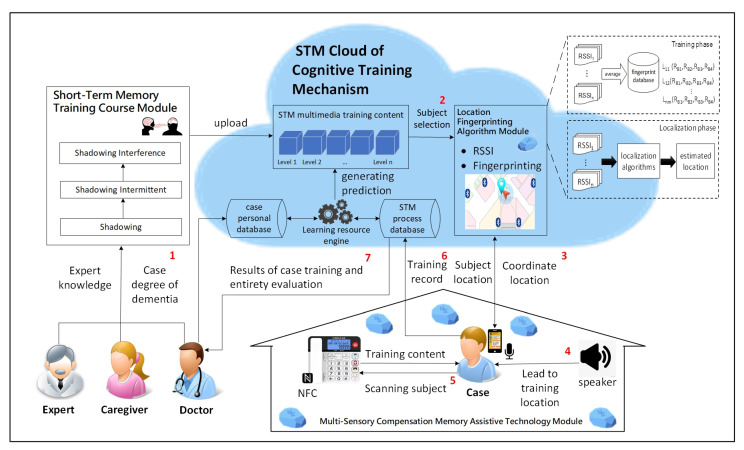
Context diagram of the digital short-term memory cognitive training system. NFC: the near-field communication

The short-term memory training model environment design of indoor positioning systems integrating physical and digital learning content is shown in Figure 5. The steps were as follows: (1) The dementia level of the patients was first assessed by a physician before they started the training (to achieve a favorable training effect). The participants trained in the study were patients with a mini-mental state examination score of ≤23 points. (2) The cloud program used basic data of the participants (e.g., age and dementia level) and personal learning history to generate appropriate multimedia training content as well as selected the target for training. (3) The fingerprinting localization calculation module detected the current location of the participants. (4) A welcome message was played, and the participants received direction through a smart speaker. (5) After the participants arrived at the training target, the system scanned the near-field communication (NFC) tag on the training object and displayed specific training content. (6) A record was saved to the cloud via a smartphone after the training was completed. (7) Professional medical personnel easily obtained the training data of a case remotely to conduct an overall assessment.

#### 3.1.1. Localization Calculation

The fingerprinting localization was divided into a training phase and a localization phase. The training phase collected the signal characteristic parameters of each RP in the required positioning area and established a fingerprint database of the correspondence between the measured RSSI and its location. The localization phase used the fingerprint database and the parameters of the received signal to match the data in the database and to identify the nearest calibration points, which were ultimately used to estimate the location of the target node.

The principle of RSSI is to estimate the distance between the transmitter and the receiver, based on the relationship between signal intensity and distance decay. The distance calculation was as in Equations (1) and (2), where *n* represents the path loss according to the communication environment and $d_{0}$ is the RSSI value of the reference distance from the receiver; a smaller value indicates a greater distance between the transmitter and the receiver. The unit is expressed in decibel milliwatts (dBm):

(1) $$RSSI\left(d\right)\;=\;-10_{n}\log_{10}\left(\frac{d}{d_{0}}\right)+RSS\left(d_{0}\right)$$

(2) $$d\;=\;d_{0}\cdot 10^{\frac{RSS\left(d_{0}\right)-RSS\left(d\right)}{10n}}$$

**Figure 6:**
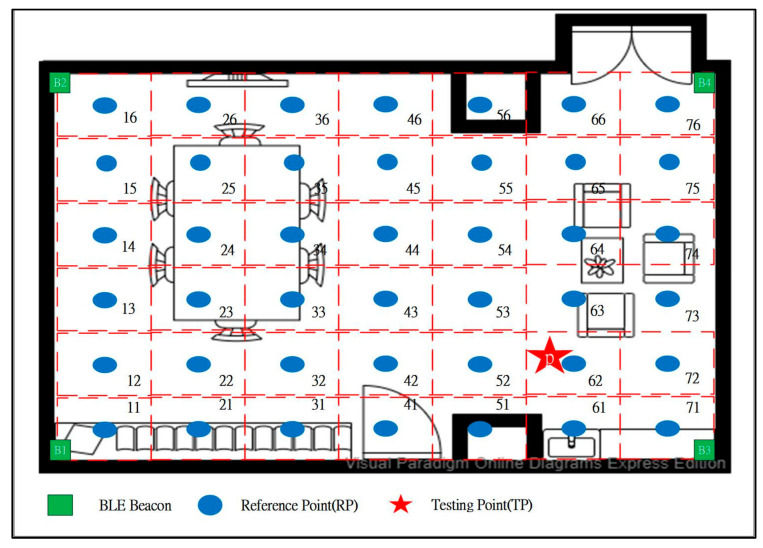
Bluetooth low energy beacon deployment context diagram.

The signals of the RPs in the experimental field were collected on the basis of Equations (1) and (2), and each RP was represented by a numbering of (xy) (as shown by the blue dots in Figure 6). $R_{\left(xy\right)}$ is the RSSI collected at the RP over a period of time. These signals were built into a fingerprint database for localization and matching purposes, as represented by a vector in Equation (3); $rssi_{ij}^{xy}$ refers to the RSSI value received from the j^th^ beacon at the xy during the i^th^ scan. The range of i is 1 ≤ i ≤ m, where m is the number of scans; the range of j is 1 ≤ j ≤ n, where n is the number of beacons.

(3) $$R_{\left(xy\right)}\;=\;\left[\alpha_{1}^{xy}\ldots \alpha_{n}^{xy}\right]\;=\;\left[\begin{array}{ccc} rssi_{11}^{xy} & \cdots & rssi_{1n}^{xy}\\ \vdots & \ddots & \vdots \\ rssi_{m1}^{xy} & \cdots & rssi_{mn}^{xy} \end{array}\right]$$

Since an indoor environment can be complex, have various sources of interference, and be prone to multipath effects, signal attenuation may be unsatisfactory. Therefore, the mean filter was used to remove the noise, as shown in Equation (4), which was mainly used to correct the signal for improved positioning accuracy. The RSSI after removing the noise is represented by the vector ${\bar{\alpha }}_{n}^{xy}$ in Equation (5):

(4) $$T\;=\;\frac{1}{n+1}\overset{n}{\underset{i\;=\;0}{\sum }}T_{i}$$

(5) $${\bar{R}}_{xy}\;=\;\left[{\bar{\alpha }}_{1}^{xy}\cdots {\bar{\alpha }}_{n}^{xy}\right]$$

However, the use of only radio frequency for positioning cannot reflect the actual position due to factors in the environment, such as diffraction and refraction. Therefore, most studies have added machine learning algorithms, such as nearest neighbor (NN), KNN, or WKNN [24,25,26], to estimate the final position and to improve accuracy. This study referred to the WKNN positioning method proposed by Fan et al. [27] and Subedi and Pyun [22] to identify the location of the participants in the environment.

**Figure 7:**
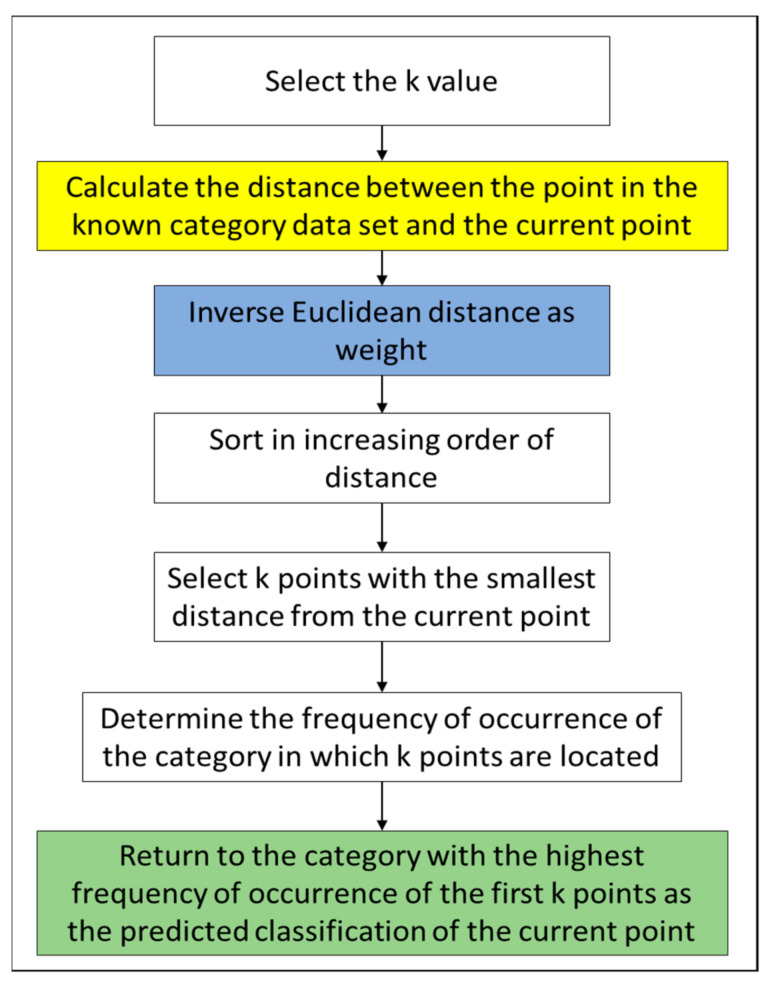
Process of the weighted K-nearest neighbors algorithm.

In the KNN algorithm, the distance between the test point and the RP must be calculated to select the closest *k* data ($y_{1},y_{2},\ldots ,y_{k}$) in estimating the final location, where $y_{1}$ represents the RP closest to the test point and $y_{2}$ is the second RP closest to the test point. A flow chart is provided in Figure 7, and a description follows.

The *k* value selection in the KNN algorithm is a parameter used to define neighbors, and the first K RPs with the smallest distance are selected as the candidate points. The model complexity is high if the K value is small, which is likely to cause over-fitting problems and increases the estimation error. By contrast, although the estimation error is reduced in the case of a larger K value, the approximation error increases, which affects the estimation results. Therefore, a smaller value is generally selected in the KNN algorithm, and the K value must be an integer. In this study, a K value of 3 was selected through experiment.

(1) Calculate the distance between the test sample vector and the RP vector (shown in the yellow block in Figure 7). The calculation method is as shown in Equation (6), where *i* is the total number of beacons in the environment and *n* is the number of selected RPs. $RSSI_{i_{online}}$ represents the coordinate of the signal value in the localization phase, and $RSSI_{i_{offline}}$ is the coordinate of the signal value received in the training phase.
  (6) $$d_{i}\;=\;\overset{n}{\underset{i=1}{\sum }}\sqrt{{\left(RSSI_{i_{online}}-RSSI_{i_{offline}}\right)}^{2}}$$
(2) Calculate the weight (shown in the blue block in Figure 7) and reciprocate the distance between the test point and the RP, as shown in Equation (7). WKNN is an improved algorithm for KNN to further improve accuracy. The intuition behind WKNN, is to give more weight to the points which are nearby and less weight to the points which are farther away [28]. In this paper, the weight is selected as the difference between the signal strength corresponding to each RP (fingerprint node) and the sum of the signal strength of each TP (Test Point) node.
  (7) $$w_{i}=\frac{1}{d_{i}^{}}$$
(3) Sort in ascending order according to distance.
(4) Select K points that have the smallest distance to the current point.
(5) Determine the occurrence frequency of the category where the K points are located.
(6) Calculate the last coordinate (shown in the green block in Figure 7), and return to the category that has the highest occurrence frequency for the first K points. This category will be the predicted classification of the current point (as shown in Equation (8), where ($x_{RP}$) is the *x*-axis coordinate value of the *i*^th^ RP and $y_{RP}$ is the *y*-axis coordinate value of the *i*^th^ RP).
  (8) $$P_{\left(x,y\right)}=\left(\frac{\sum_{i=1}^{K}\left(x_{RP}\times w_{i}\right)}{\sum_{i=1}^{K}\left(w_{i}\right)},\frac{\sum_{i=1}^{K}\left(y_{RP}\times w_{i}\right)}{\sum_{i=1}^{K}\left(w_{i}\right)}\right)$$

#### 3.1.2. Content-Driven Process Design of the Fixed-Point Interactive Course 

**Figure 8:**
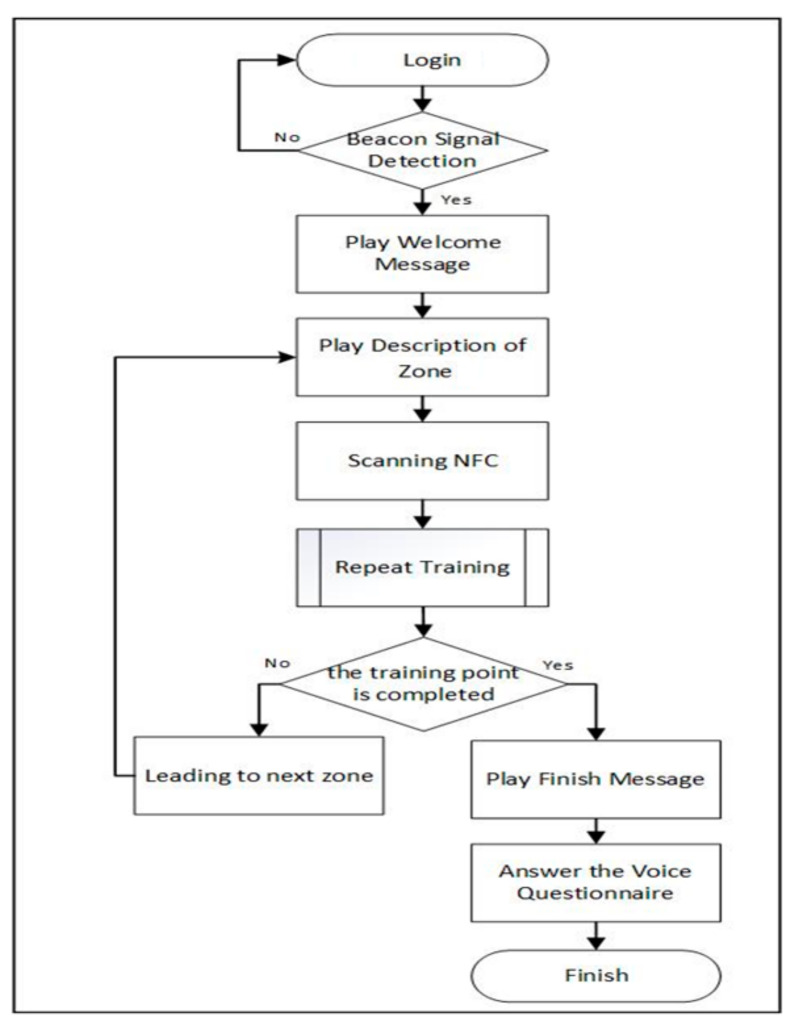
Digital cognitive training flow chart.

The system calculated the location points according to the positioning calculation process to drive the short-term memory training process of physical and digital learning. The so-called “interactive” model was to provide specific content to the participants according to the personal identity (ID) and the medication order of the user. The process is depicted in Figure 8. First, the participant used a specific account to log in to the application and received the beacon signal in the environment through the mobile phone. A welcome message was played if the receiver received the beacon signal location point set in the experiment. Subsequently, operating instructions were provided according to the area the participant was located, and the participant was guided to scan the NFC tag. After scanning, the shadowing exercise was begun. The process was interactive through voice. After the operation, the system guided the participant to the next training point according to the training prescription in the database until all training points were completed. Finally, the participant was guided to the cloud voice performance evaluation point to complete a five-minute Mandarin voice survey. During the operation, the system recorded the user ID, NFC tag ID, and the start and end time of the course in the backend for viewing and analysis by medical personnel.

### 3.2. Shadowing System

The digital shadowing system can be divided into a frontend memory training application content design and a backend management platform. A multi-sensory learning environment was constructed through the IoT and a software platform. Multi-sensory learning allowed the participants to use digital voice learning materials and physical operation (e.g., smartphone and remote control) for training. The inclusion of multiple memory types, including auditory, visual, and haptic stimulation, can improve memory performance [29]. This section explains the design concepts of the short-term memory training content provided in the study and introduces the management platform.

#### 3.2.1. Training Content Design

The design of short-term memory cognitive training courses for dementia was based on the principles of safety, simplicity, and challenge. Only by reducing the generation of negative emotions in the older adults can the effectiveness of cognitive training and the motivation to participate in activities be improved. Because the degree of degeneration of each patient differed, assistance from professionals was required to make adjustments and confirm the needs and goals of the patients, as well as to provide strategies and information to assist in the use of compensatory memory aids to prevent cognitive decline and social withdrawal situations and thus delay dementia [30].

The content of the digital shadowing exercise designed for this study was based on the shadowing and shadowing with a twist methods [12], combined with the memory strategy proposed by Sternberg and Sternberg [11], and was discussed and revised with clinical physical therapists. The digital shadowing exercise model can be divided into three parts—shadowing, shadowing with a twist, and shadowing with interference—to train participant cognitive functions in the process. Each part of the training was divided into three modules of easy, medium, and hard, which allowed the participants to train from simple to difficult stepwise. In addition, daily activity events and related experiences (e.g., emergency telephone number 119, mobile phone number, and recent visit time) were added to the content. The training steps were as follows:

(1) The participant logs into the application.
(2) The participant scans the NFC tag.
(3) The application reads a number series.
(4) The participant shadows the content.
(5) The application plays interference sounds of a car and a butterfly (shadowing with interference).
(6) The participant shadows the interference sounds (shadowing with interference).
(7) The participant operates the physical objects.
(8) The participant says “confirm” upon completion of the operation.

Upon completion, the system wrote the operating time into the database as the basis for assessment by professionals and the adjustment of subsequent courses. This set of applications was developed for the Android 8.0 system by using the Java programming language. All training commands were interactive with text-to-speech and automatic speech recognition technologies. The participants controlled the learning steps at their own pace.

#### 3.2.2. Management Platform of the Cognitive Learning Course 

The purpose of developing a learning management system was to provide case managers with an effective management tool to continuously track the progress and performance of patients in various training and learning activities. The development tool used Apache (The Apache Software Foundation, Wilmington, DE, U.S.A.) as the main server software and MySQL as the database engine. The web backend pages were designed using JavaServer Pages, and the frontend was designed with a combination of HTML, JavaScript, and CSS.

**Figure 9:**
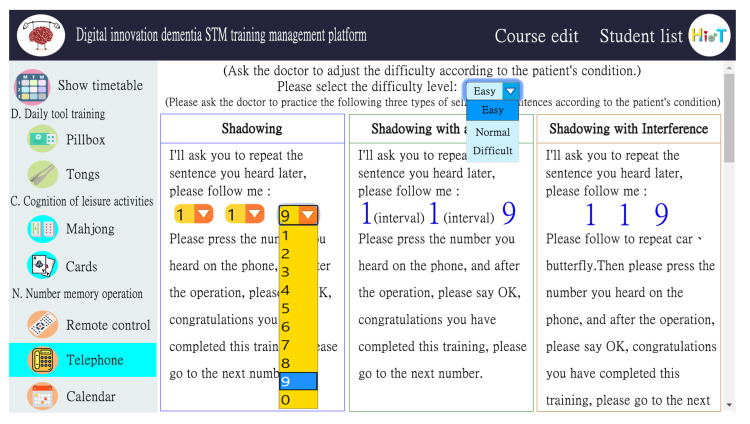
Digital short-term memory cognitive training content management platform.

The innovative, digital short-term memory training management platform for dementia is usable on any browser to facilitate content management by medical personnel. Figure 9 depicts the course editing interface. The left half of the screen is a list of various objects. After clicking on a single object, the user can edit the training content for that object. The right half of the screen is the training content of a single object; listed from top to bottom are shadowing, shadowing with a twist, and shadowing with interference. A case manager is able to select difficulty levels through a drop-down list to edit and update the content.

**Figure 10:**
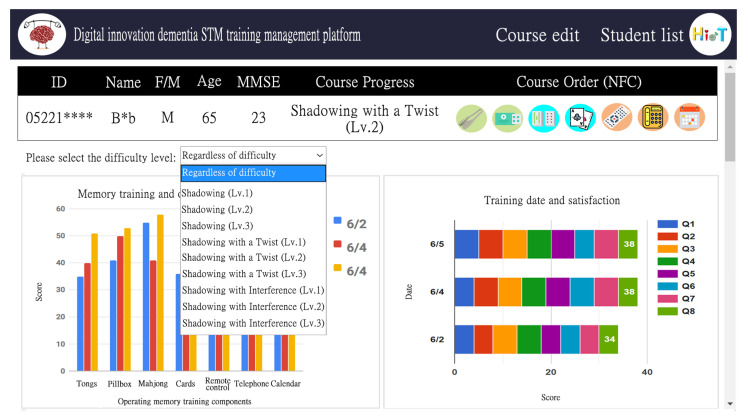
Statistical diagram interface of digital short-term memory cognitive training results.

The platform also contained a learner list management function. Compared with conventional cognitive training activities, the digital cognitive training system is more efficient in quantifying the training data and served as a tool for remote monitoring and cloud management by medical personnel; thus, case managers can accurately grasp the progress of patients. As shown in Figure 10, the left side is a histogram of the relationship between memory training and the operation time of each training item. Dates are distinguished by color, and records of up to one month are displayed. In addition, the training situation for different courses can be selected through a drop-down list. The information is presented through graphs so that professional medical personnel can observe the overall training situation of participant short-term memory. The right side of Figure 10 is a stacked bar chart of the relationship between training date and satisfaction score. The color represents the score of each question; moving the cursor over a bar provides the score for a question.

## 4. Results

### 4.1. Experiment Simulation for Positioning

**Figure 11:**
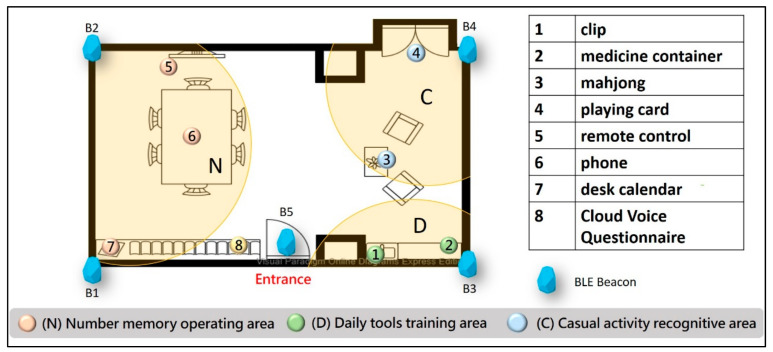
Deployment points of digital short-term memory cognitive training materials.

This study was conducted in a professional medical environment. A beacon signal intensity test was performed to identify the most appropriate deployment point and to solve positioning error problems such as signal interference and drift in the indoor positioning method. The experiment used beacons deployed in the environment to divide the space into the following areas: the starting point, number memory operation (N), daily tool training (D), and cognition of leisure activity (C). Two beacons B1 and B2 were deployed in area N, B3 was deployed in area D, and B4 was deployed in area C, with seven training points and one cloud voice performance evaluation point in each area, as shown in Figure 11.

#### Simulation Results of Positioning Calculation

**Figure 12:**
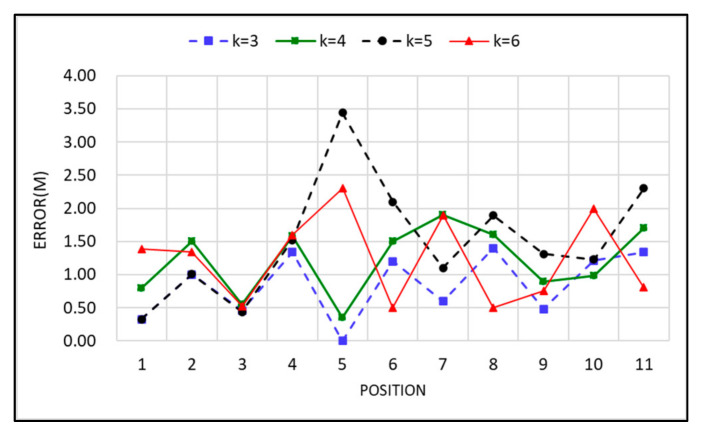
Positioning error result.

The experiment was conducted in a 48 m × 32 m plane space to evaluate the positioning accuracy of the fingerprint positioning algorithm method. The frontend positioning equipment used Bluetooth 4.0 beacons as signal transmitters and an Android mobile phone as the receiver. Figure 12 illustrates the mean positioning error results measured using 42 RSSI vectors with different *k* value parameters. The experimental results indicated that the mean error was approximately 2 m, and *k* = 3 was the minimum standard deviation. Therefore, *k* = 3 was used for the experiment.

**Figure 13:**
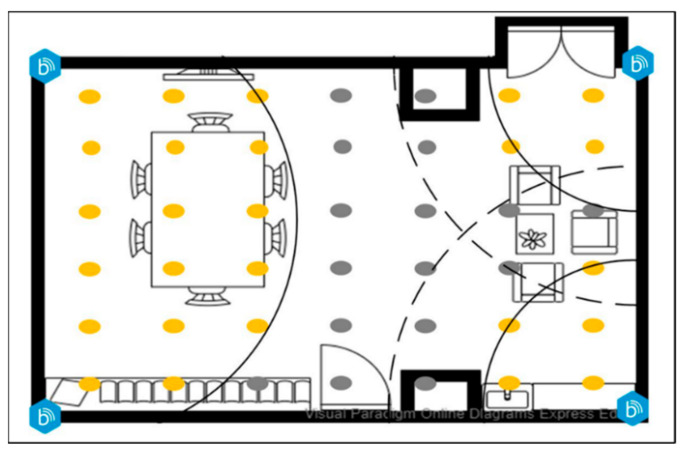
Active zone scope definition.

Regarding the experimental design, in order for the participant to perform the training correctly, they must receive the learning content at the active zone and then start training. When the participant started the training mode, the beacon in the environment and the RSSI value received by the mobile phone were compared using the fingerprint positioning algorithm. When the participant was located in the default active zone at the backend of the system, the user ID and the beacon media access control address were used as the primary key to search for the corresponding data in the database, and information from the cloud server was transmitted to the smartphone. The active zone was defined according to the signals collected during the training phase. Ideally, each active zone signal would be independent without overlap. Nonetheless, B3 and B4 partially overlapped in the experiment (as shown in the dashed intersection range in Figure 13), which may cause misjudgment. The range of the circle center must be narrowed until no overlap occurs (the solid line range in Figure 13). Therefore, the boundary value of the active zone was defined to be reduced to −55 dBm to −65 dBm, and the area the participant was located was established.

### 4.2. Validation and Assessment Test

The proposed training mechanism was to develop virtual and physical integrated, digitized cognitive training content to provide patients with MCI appropriate training stimulation, and to understand the training of each patient through systematic and continuous recording. To examine the performance of the system, we conducted functionality and acceptance testing as part of the system development process. The specific objectives of the pilot test included:

(1) Understanding the user’s satisfaction, ease of use, and feedback with regard to the system for possible future improvements, and
(2) Understanding physician views of the system.

#### 4.2.1. Participants

The pilot test was conducted from May to June 2020 in Taipei City. Participants with mini-mental state examination scores less than or equal to 23 points, normal vision and hearing, sufficient cognitive ability, and the ability to understand sentences were recruited. The sample included 10 participants with mild dementia (five male and five female individuals). Basic information for the participants is provided in Table 1. Two participants withdrew due to personal factors, and eight participants completed the experiment.

During the study period, the participants received two training sessions per week over 5 weeks. They were required to complete the training course for that day in accordance with the course list of the system command and to answer a 5-min Mandarin voice satisfaction questionnaire after the session. The questionnaire included questions (one open-ended) about their experience in using the hardware, software, and applications and about their satisfaction with the hardware and the system. In addition, the participants were required to complete a System Usability Scale (SUS) in week 5.

#### 4.2.2. Participant Responses and Scale Results

**Figure 14:**
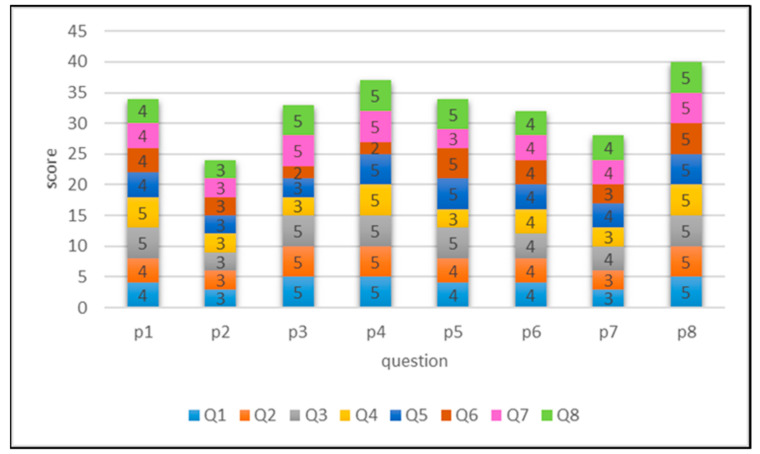
Stack graph of satisfaction score survey results (P1—8 are participants and Q1—8 are questions).

The interactive voice satisfaction questionnaire was designed to understand participant acceptance of the system, with a total of eight closed questions and one open-ended question. The closed items were measured using a five-point Likert scale, with a maximum score of 40 points; higher scores indicated greater satisfaction. The mean score for the survey was 4.093 points (the results for each question are shown in Figure 14). Question 3, “The equipment or device used in the training course is easy to operate” was scored highest (mean 4.5 points), which suggested that the equipment used in the training course was convenient for the participants to operate. According to the survey results, all the participants except participant P4 had experience using a smartphone, thus, the use of digital tools in cognitive training was an acceptable approach for most. By contrast, Question 6, “Participating in this training course is helpful to my current situation” was scored lowest (mean 3.5 points), probably because of poor course content design or an inadequate number of participations. Since cognitive impairment involves various aspects, qualitative research was performed on topics with poor scores, and the design and training of related activities were revised. More training items can be developed in the future. In addition, the satisfaction scores of participant P2 were the lowest among the participants (mean 3 points). In an in-depth interview, P2 stated that the number of participations was insufficient to allow him to determine whether the training course was helpful, and the system operation instructions were unclear.

An international SUS was used to test the usability of the system. This scale was proposed by Brooke in 1996 [31]. It has a Cronbach’s α value of 0.802 and has excellent internal consistency [32], making it suitable for evaluating various products and services, including hardware, software, mobile devices, websites, and applications. It employs a five-point Likert scale and contains a total of 10 questions (five positive and five negative). The score ranges from 0 to 100 points. A higher score indicates higher system usability.

For the SUS test results, the mean, median, maximum, minimum, and standard deviation for the participants were 62.8, 63.75, 75, 50, and 11.05 points, respectively. Inferred from the highest and lowest scores and the high standard deviation, the main effect may be influenced by the difference between the cognitive impairment degree and aspects of the participants. Moreover, the mean score was low. In addition to personal factors, certain participants responded that they could not fully understand the items on the questionnaire, which led to inaccurate responses.

**Figure 15:**
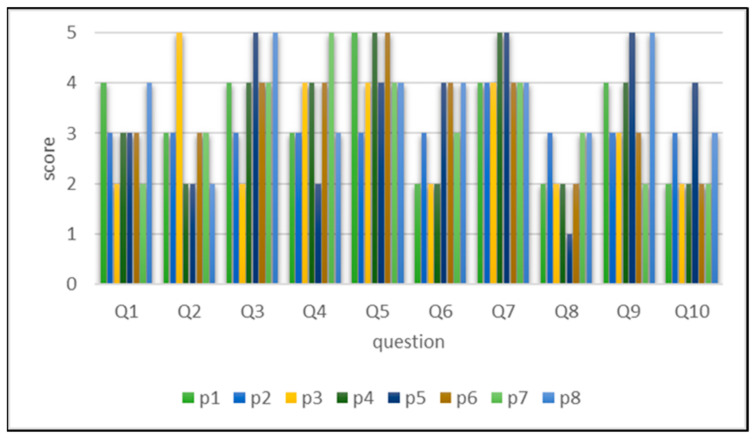
Statistical results of the System Usability Scale.

The statistics of the participant answers are presented in Figure 15. Among the positive questions, Question 5, “I think the functions of this system are well integrated” and Question 7, “I think most people can quickly master the use of this system” had the highest mean scores (both 4.25 points). This finding indicates that the participants agreed that the system was easy to operate, and its function integration was also satisfactory. In addition, the participants felt that the integration of physical and digital technology did not require much learning time and were happy with the process. By contrast, the lowest score was for Question 1, “I think I will use this system often,” with a mean score of 3, signifying that the participants were reluctant to use the system regularly. In in-depth interviews, some of the participants responded that they did not want to perform training twice in a day because this caused difficulty in concentrating.

Among the negative questions, Question 4, “I need the assistance of a technician to use this system” received the highest score, with a mean of 3.5 points. In interviews, most of the participants said that they needed the assistance of technicians when operating the system for the first time. With the assistance of technicians, the entire training process was smoother, and they were more confident in completing the course tasks. By contrast, the lowest score was for Question 8, “I think this system is troublesome to use,” with a mean score of 2.25 points; thus, most participants did not think that using this set of training courses was troublesome.

#### 4.2.3. Overall Response from Doctors

The responses of three physicians regarding the availability and the quality of the system were as follows. Doctor 1 stated: “Cognitive training is a more targeted treatment method. Most of the cognitive training content currently provided in clinical practice relies on case managers and occupational therapists to design content for each patient based on their own learning and experience. However, cognitive training requires standardized design, which is suitable to constructing in an information-based approach.” The purpose of constructing the system was to use information and communication technology to establish a cognitive training mechanism allowing patients to be trained systematically, and this point was raised by Doctor 1. Doctor 2 said: “As a neurologist, I hope that patients will have the opportunity to have access to cognitive training so that they can stabilize the disease development under the medical treatment and nonpharmacological management. This system not only allows the patients to train independently outside the hospital but also allows me to prescribe training remotely according to their training progresses.” Doctor 2 thus highlighted the importance of cognitive training outside the hospital and the convenience of the system. Doctor 3 commented: “With the aid of this system, I can check patient training records online to understand their current conditions and provide them with appropriate medical services.” Doctor 3 thus described how the updated records in the system allow him to understand patient cognitive statuses, and he could provide appropriate medical services.

## 5. Conclusions

This study developed a digital multimedia cognitive training course mechanism for patients with mild dementia. The clinical collective training mode was dispersed to individualized training, which allowed the patients to have more opportunities for autonomous practice through a process integrated with the physical environment without assistance from others. A Bluetooth indoor positioning system and short-range wireless communication were used to provide the participants with multi-sensory stimulation and rehearsal training. The results indicate the learning effects of cognitive training were primarily achieved. Such training is expected to enable older adults to retain autonomy in daily life and to create a self-help, positive self-esteem environment for them.

A preliminary assessment was conducted, particularly in terms of usage satisfaction and technology acceptance. The system performed well in satisfaction feedback (mean score of 4.093 points). However, regarding system usability, more encouragement and interaction were needed to actually reduce the time necessary for caregivers or technicians to assist the participants to complete training courses independently. In sum, however, compared with other interventional tools, the digital cognitive training intervention developed for this study was practical for patients with MCI and did not incur prohibitive costs. The proposed system can provide a reference for future clinical digital smart nonpharmacological management and achieved the goal of allowing observation of activities of daily living of patients with dementia. However, it is important to note that our testing is preliminary and limited given the purpose of the system testing and the small sample size. We plan to conduct a comprehensive and in-depth study for future work.

The training course content designed for this study can be revised, and two specific suggestions are made to improve the model. First, multiple signal overlaps and interferences occurred because the measurement environment was contained in a small space. Positioning accuracy and associated benefits can be greatly improved if the study is implemented in a larger space. In addition, the training process was designed mainly to measure the smooth completion of each stage. Therefore, participants were required to complete all the tasks simultaneously in each training session. Although the preliminary results received excellent satisfaction feedback, medical personnel were required in the long-term to establish different training prescriptions and assess difficulties for each patient. By that time, the training path of each participant will be different during the activity. In terms of longitudinal observation and recruitment, this method is more likely to strengthen the specific contribution of the innovative application of the indoor positioning system to short-term memory cognitive training. Second, the assessment was essentially concerned with the ease of use and usability of the system. However, the brevity of the training program meant that the researchers were unable to observe whether the intervention tools were highly positive and significant for the current situation of the participants. Future studies with a longitudinal research design are recommended to track changes in short-term memory and to explore the empirical effects of the memory training intervention.

## Figures and Tables

**Table 1:** Details of the patient sample.

Patient	Age	Gender	Education	Mini-Mental State Examination
**P1**	64	M	elementary	23
**P2**	69	M	junior	22
**P3**	77	F	junior	22
**P4**	69	F	senior	20
**P5**	65	M	junior	23
**P6**	73	M	college	22
**P7**	65	F	junior	23
**P8**	69	F	senior	23
**P9**	70	F	elementary	21
**P10**	71	F	elementary	20
**Total Number (TN)/Average(AVG)**	69.2(AVG)	5 M, 7 F(TN)	3 elementary, 4 junior, 2 senior, 1 college(TN)	21.7(AVG)

## Data Availability

Not applicable.
